# Food systems for sustainable development: proposals for a profound four-part transformation

**DOI:** 10.1007/s13593-018-0519-1

**Published:** 2018-08-09

**Authors:** Patrick Caron, Gabriel Ferrero y de Loma-Osorio, David Nabarro, Etienne Hainzelin, Marion Guillou, Inger Andersen, Tom Arnold, Margarita Astralaga, Marcel Beukeboom, Sam Bickersteth, Martin Bwalya, Paula Caballero, Bruce M. Campbell, Ntiokam Divine, Shenggen Fan, Martin Frick, Anette Friis, Martin Gallagher, Jean-Pierre Halkin, Craig Hanson, Florence Lasbennes, Teresa Ribera, Johan Rockstrom, Marlen Schuepbach, Andrew Steer, Ann Tutwiler, Gerda Verburg

**Affiliations:** 1 Université de Montpellier (Université Montpellier), Montpellier, France; 2 CIRAD, DGDRS, 34398 Montpellier, France; 3 Ministry of Foreign Affairs and Cooperation, Torre Norte, Planta 1. Serrano Galvache, 26, 28071 Madrid, Spain; 4 Imperial College, South Kensington Campus, London, SW7 2AZ UK; 5 CIRAD, DG, 34398 Montpellier, France; 6 Agreenium, 42 rue Scheffer, 75116 Paris, France; 7 IUCN, Rue Mauverney 28, 1196 Gland, Switzerland; 8 Institute of International & European Affairs, 8 N Great George’s St., Rotunda, Dublin, Ireland; 9 IFAD, Via Paolo di Dono, 44, 00142 Rome, RM Italy; 10 Ministerie van Economische Zaken en Klimaat, Ministerie van Infrastructuur en Milieu, The Hague, The Netherlands; 11 Economic Council on Planetary Health, Oxford Martin School, 34 Broad St, Oxford, OX1 3BD UK; 12 NEPAD Agency, 233 15th Road, Midrand, South Africa; 13 RARE, 1310 N. Courthouse Road, Ste. 110, Arlington, Virginia 22201 USA; 14 CCAFS, CGIAR, Wageningen University and Research, 6708 PB Wageningen, The Netherlands; 15 Climate Smart Agriculture Youth Network, SDG Action Campaign, P.O. Box, 8860 Yaounde, Cameroon; 16 IFPRI, 1201 Eye St., NW, Washington, DC 20005-3915 USA; 17 UNFCC, UN Campus, Platz der Vereinten Nationen 1, 53113 Bonn, Germany; 18 CCAFS Program Management Unit, Wageningen University & Research, 6708 PB Wageningen, The Netherlands; 19 Department of Foreign Affairs and Trade, Iveagh House, 80 St. Stephen’s Green, Dublin, Ireland; 20 European Commission, 200 rue de la Loi, 1049 Brussels, Belgium; 21 World Resources Institute, 10 G Street NE Suite 800, Washington, DC 20002 USA; 22 Agronomist, 8 Chemin du Gué, 01210 Ferney-Voltaire, France; 23 IDDRI, 41 Rue du Four, 75006 Paris, France; 24 Stockholm Resilience Centre, Stockholm University, Kräftriket 2B, SE-10691 Stockholm, Sweden; 25 Scaling Up Nutrition Movement, Palais des Nations, CH-1211 Geneve, Switzerland; 26 Bioversity International, Via dei Tre Denari 472/a, 00057 Maccarese (Fiumicino), Rome, Italy

**Keywords:** Food systems, Agriculture, Transformation, Nexus, Sustainable development, Climate change, Koronivia

## Abstract

Evidence shows the importance of food systems for sustainable development: they are at the nexus that links food security, nutrition, and human health, the viability of ecosystems, climate change, and social justice. However, agricultural policies tend to focus on food supply, and sometimes, on mechanisms to address negative externalities. We propose an alternative. Our starting point is that agriculture and food systems’ policies should be aligned to the 2030 Agenda for Sustainable Development. This calls for deep changes in comparison with the paradigms that prevailed when steering the agricultural change in the XXth century. We identify the comprehensive food systems transformation that is needed. It has four parts: first, food systems should enable all people to benefit from nutritious and healthy food. Second, they should reflect sustainable agricultural production and food value chains. Third, they should mitigate climate change and build resilience. Fourth, they should encourage a renaissance of rural territories. The implementation of the transformation relies on (i) suitable metrics to aid decision-making, (ii) synergy of policies through convergence of local and global priorities, and (iii) enhancement of development approaches that focus on territories. We build on the work of the “Milano Group,” an informal group of experts convened by the UN Secretary General in Milan in 2015. Backed by a literature review, what emerges is a strategic narrative linking climate, agriculture and food, and calling for a deep transformation of food systems at scale. This is critical for achieving the Sustainable Development Goals and the Paris Agreement. The narrative highlights the needed consistency between global actions for sustainable development and numerous local-level innovations. It emphasizes the challenge of designing differentiated paths for food systems transformation responding to local and national expectations. Scientific and operational challenges are associated with the alignment and arbitration of local action within the context of global priorities.

**Contents**

- 1. Introduction
- 2. Food systems: an integrated perspective to address the “food and nutrition security, ecosystem integrity, climate and social justice” nexus
- 3. Food systems transformation for sustainable development: the four parts
  3.1. Healthy and sustainable food consumption patterns
  3.2. A new vision of sustainable agricultural production and food value chains
  3.3. Contributing to mitigate climate change
  3.4. A renaissance of rural territories
- 4. The new food systems transformation
  4.1. Assessing the contributions of food systems to the SDGs
  4.2. Achieving impact at scale through local-level action
  4.3. Managing the intersection of global and local priorities through territorial approach
- 5. Conclusion
  References

## Introduction

An exceptional process reached its conclusion in 2015. For the first time in history, world leaders have unanimously agreed on a vision for the future of humanity: the 2030 Agenda for Sustainable Development. Through a set of 17 Sustainable Development Goals (SDGs) and 169 targets (UN 2015), the agenda articulates a universal and integrated plan of action of application in all countries, developed and developing alike. The 2030 Agenda integrates the three dimensions of sustainable development across the 17 SDGs, and within each of the goals, together with human rights, peace, security, and governance. In the words of the then United Nations Secretary General, it represents a paradigm shift and a plan of action for dignity, people, planet, prosperity, justice, and partnerships (UN Secretary General, 2014*.* paragraph 64). In this framework, SDG 2 aims to “End hunger, achieve food security and improved nutrition, and promote sustainable agriculture,” while SDG 13 urges to “Take urgent action to combat climate change and its impacts.” The impact of climate change undermines human rights and reinforces inequalities and injustice. In this way, climate action is also a moral imperative that brings justice to the center of the climate-poverty-development discussion, a message that is at the core of Pope Francis’ Encyclical “Laudato Si” and the Climate Justice perspective (Robinson 2015). Through the Paris Agreement on climate, 195 countries have established a universal action framework in line with the 2030 Agenda for Sustainable Development (Nature Climate Change 2016). The SDGs set concrete targets for multiple issues and sectors that are critical to climate action.

Against this backdrop, the then UN Secretary General Ban Ki-moon convened an informal High-Level meeting of experts and policy-makers in Milan on the 2015 World Food Day (“Milano Group”), with the mission of laying out shared views on the following: (i) a strategic narrative that links climate, agriculture, and food, (ii) emerging opportunities for bringing this narrative to the climate debate, and (iii) options for action. This paper builds on the outcomes of the Milano Group’s deliberations and focuses its main conclusion: the need for the transformation of food systems—at scale—in order to achieve the SDGs and the Paris Agreement. The transformation should deliver multiple and simultaneous social, economic, and environmental outcomes, including poverty eradication and mitigation and adaptation to climate change. This consensus implies a radical shift in comparison with the paradigms that steered the agricultural changes of the XXth century. We therefore refer to a new transformation in food systems, in agriculture, and in rural livelihoods.

After examining the links between agriculture and food and nutrition security (FNS) and the evolution of the role of agriculture for development, we conclude with the need to move beyond food supply as the basis for food systems. We identify four essential parts for the transformation of all food systems. We also discuss some of the principles that should underpin the transformations, as well as major challenges with implementation.

## Food systems: an integrated perspective to address the “food and nutrition security, ecosystem integrity, climate and social justice” nexus

The sustainable development of the world’s people and of their planet is only possible if all people are food secure and well-nourished, if all ecosystems are healthy and balanced, if societies are resilient in the face of threats posed by climate change, and if governance of development benefits is fair and just. Food systems “consist of all the elements (environment, people, inputs, processes, infrastructures, institutions, etc.) and activities that relate to the production, processing, distribution, preparation and consumption of food, and the outcomes of these activities” (HLPE 2014).

Agriculture and fisheries are the primary livelihoods for most of the world’s people and influence all these realities. One can easily understand the exclusive focus and pressure placed on the agricultural sector by the injunction to “produce more” over the past two centuries. It was no easy task to enable the exponential growth of the global population, moving from 1 to 7 billion people in two centuries and from 3 to 7 billion between just 1960 and 2010, while Malthus observed a linear increase in agricultural production (Malthus 1798). Wars and famines were avoided, and the prophecy of Malthus was not fulfilled—thanks to the Green Revolution. While population doubled between 1961 and 2003, global food production increased by a factor of 2.5 (Paillard et al. 2011), leading to a steady increase in the average food available per person, from 2373 kcal/person/day in 1969/71 to 2772 kcal/person/day in 2005/07 (FAO 2012). This increase in production was associated with significant changes in food systems with major risks to food security confined—in the main—to localized populations affected by violent conflict and/or unexpected weather events. However, there are underlying risks associated with a “high level of corporate concentration in food trade, transformation and distribution” (HLPE 2017a), unequal endowments in agricultural assets, difference in access to natural resources (De Schutter 2011), and inequalities in people’s income.

Agriculture has suffered from a lack of public interest and investment in recent decades. As a consequence of the riots that affected many countries in early 2008 due to the spike in food prices, agriculture was back on center stage in the scientific literature (Godfray et al. 2010; Guillou and Matheron 2014) and in the political agenda (HLTF 2008; reform of the Committee on World Food Security; priority in the G20 and G8 agendas). Evidence shows that global and regional per capita availability of food has constantly increased during recent decades and that the available global supply was not the basis of this food crisis. Rather, it was inequalities of access to food because of extreme differences in people’s purchasing power and excessive trade-related volatility in world food prices. This was a result of an erosion of planning and regulatory capabilities at every level: the consequence was a global crisis of the food system (Headey 2011) that threatened the global economy and drove political instability throughout the world.

However, the boost in attention that resulted from the 2007–2008 food price crisis (MC Arthur 2015) has not led to a sustained increase in the level of political attention given to agriculture and food systems. This is a paradox given that well-functioning food systems are critical for advancing the 2030 Agenda for Sustainable Development. Hence, there is a need for new narratives and better means for their communication, starting with an explanation of why food systems are so important. Firstly, agriculture and fisheries are the primary means of income for most of the world’s poor and vulnerable people (IBRD/World Bank (The International Bank for Reconstruction and Development/World Bank), 2007). Secondly, food and nutrition insecurity, as well as rural poverty, are root causes of political instability, conflict, violence, and migration (FAO 2016). Indeed, the HLPE (HLPE 2017a) reports that “unequal access to food is… a driver of many other inequalities and instability… and (leads to) to low levels of investment in the provision of public goods and services.” Thirdly, agricultural practices are highly connected to environmental health, management of natural resources, and climate change (Smith 2013). Fourthly, the crop, livestock, and fish sectors are resource intensive. They use 70% of freshwater resources (Kabat 2013) and are responsible for around 30% of total energy demand (FAO 2011a). Fifthly, agriculture is at least twice more effective than any other sector in reducing poverty (IBRD/World Bank 2007, *op. cit.*) and will continue to play a pivotal role in efforts to reduce extreme poverty (Christiaensen et al. 2011). Since agriculture is—worldwide—the main source of jobs (30.7% of the world’s workers were employed in the agriculture sector in 2014; FAO 2015a), the rural sector contributes to around half of the total reduction in extreme poverty (de Janvry and Sadoulet 2010: p. 18).

The agriculture sector has only recently given priority to climate change, in particular to its increasingly dramatic impact on the millions of small-scale family farmers and food processors. This is significant as they produce around 80% of the food consumed in the world (Sourisseau 2015; IAASTD 2009) and represent more than 80% of the 570 million households living from agriculture (Lowder et al. 2016). Climate therefore threatens the food and nutrition security of people living in the most vulnerable ecosystems (Campbell et al. 2016): this is unjust and contributes both to suffering and to forced migration. It threatens both peace and security. At the same time, the agricultural sector is a major contributor to greenhouse gas (GHG) emissions. It is directly responsible for 14% of emissions and contributes 24% if related land use changes are taken into consideration (IPCC 2014). At the 17th Conference of the Parties of the United Nations Framework Convention on Climate Change (UNFCCC) (COP17) in 2011 in Durban, the conference requested its Subsidiary Body for Scientific and Technological Advise (SBSTA) to consider issues related to agriculture. The landmark 2015 Paris Agreement has subsequently underscored the importance of ensuring food security for all: the Parties recognized “the fundamental priority of safeguarding food security and ending hunger, and the particular vulnerabilities of food production systems to the adverse impacts of climate change.” However, Article 2 of the Agreement reflects the potential trade-off between fostering low GHG emissions and ensuring that sufficient food is available for all people. This paradox is unsurprising. It reflects some of the difficulties observed in successive negotiations at the UNFCCC (Campbell 2014) and results from well-developed national positions (Caron and Treyer 2016) related to people’s food and nutrition security, to the organization of international trade, and to the need for increases in agricultural productivity. However, the need for attention both to agricultural practices and to land use is clearly identified in the vast majority of Nationally Determined Contributions (NDC; Thornton et al. 2017) to reducing greenhouse gas emissions. This reflects the capacity of changes in agriculture to contribute to climate change mitigation as well as to enable food producers to adapt to new weather patterns. The particular relevance of agriculture for adaptation is also reflected in the context of the National Adaptation Plans (NAPs) alongside the NDCs, where agriculture is a prime consideration. The 23rd conference of the parties in Bonn, 2017, has reflected the political will of parties to intensify work on agriculture with adopting the Koronivia joint work on agriculture by which the COP requests both SBSTA and SBI to jointly address issues related to agriculture (4/CP23). The recent Koronivia decision recognizes and highlights the additional challenge of achieving food security under a changing climate and specifically addresses vulnerabilities in the agriculture sector.

Hence, there are many reasons why it would not be correct to continue addressing Food and Nutrition Security solely as a global supply issue (Fouilleux et al. 2017). Population growth is no longer the main driver of demand in agriculture and food systems. Increasing per capita incomes, cash-cropping, urbanization, and changing dietary preferences are exerting ever stronger influences (HLPE 2016). Despite the need to boost food production in certain regions, most importantly in Sub-Saharan Africa, the world is not currently suffering an overall food shortage. When it identified critical and emerging issues in 2017, the HLPE highlighted the need for transformation of both production and consumption patterns and the organization of food systems. It also focused on the challenge of social and economic inequalities and the suffering of small-scale food producers and processors, especially women, who have tended to be left behind by initiatives geared to increasing production (HLPE 2017a, *op. cit.*).

There is no reason for the future to reflect the past (Paillard et al. 2009, *op. cit*.). There are numerous reasons why the evolution of food systems should shift from an exclusive focus on boosting production so as to increase the supply and availability of food. Future generations will be better served if such food systems are designed so that they contribute to achieving the SDGs. The focus of food systems should be on eradicating poverty, increasing resilience, ensuring people’s food and nutrition security, promoting good nutrition and health, reducing inequalities, contributing to peace, promoting political stability, regenerating ecosystems, and mitigating climate change. The full diversity of food systems must be taken into account as they are redesigned (Ingram 2011): the starting point must be to shift the focus from “feeding people” to “enabling people to nourish themselves” and doing this in a way that is sustainable (Haddad et al. 2016).

The overall purpose is for agriculture and food systems’ to make the greatest possible contribution to achievement of the SDGs: food systems transformation should reflect a consensus on pathways to be pursued and their potential impact—in terms of environmental, social, nutrition, and health outcomes.

## Food systems transformation for sustainable development: the four parts

Food systems provide a powerful lever for economic and social development. Agriculture, food processing, and distribution have evolved substantively in the last century because of urbanization, mechanization, and modernization. Their performance has deeply transformed most economies.

The evolution has involved the industrialization of processing, commoditization of all types of food, globalization of markets, increases in distant exchanges, and reorganisation of distribution. Even if such changes have touched only part of the agriculture sector, the dynamic that has been generated is very strong. The challenges faced by farmers, especially small- and medium-sized landholders, have been highlighted: appropriation of biological resources (Godfray et al. 2010, *op. cit.*), land tenure and grabbing (HLPE 2011b; www.landmatrix.org), increased competition, exclusion linked to standards and specifications (Reardon et al. 1999), market instability and excessive price volatility (HLPE 2011a), reduced access to credit, dismantling of support mechanisms and services (IBRD/World Bank 2007, *op. cit.*), growth and emergence of risks—particularly climate (Beddington et al. 2012), and emerging diseases (Morand and Figuié 2016).

The evolution of food systems has brought unprecedented increases in production and wealth, but many concerns have emerged regarding externalities. This has led to questions about the long-term sustainability of current agriculture and food production. They include—firstly—concerns about environmental issues and more specifically to threats regarding species diversity, ecosystem integrity, and ecosystem based services (Conway 1997; Steffen et al. 2015; Maxwell et al. 2016), as well as to related trade-offs (Phalan et al. 2011; Byerlee et al. 2014). Secondly, there are concerns about rural impoverishment, vulnerability, and human rights (Pingali 1993) which call for attention to dependency on imported food, technologies, or inputs, to health impacts of inappropriate food consumption, and to risks linked to concentration of food processing and of distribution chains (Murphy et al. 2012).

**Fig. 1:**
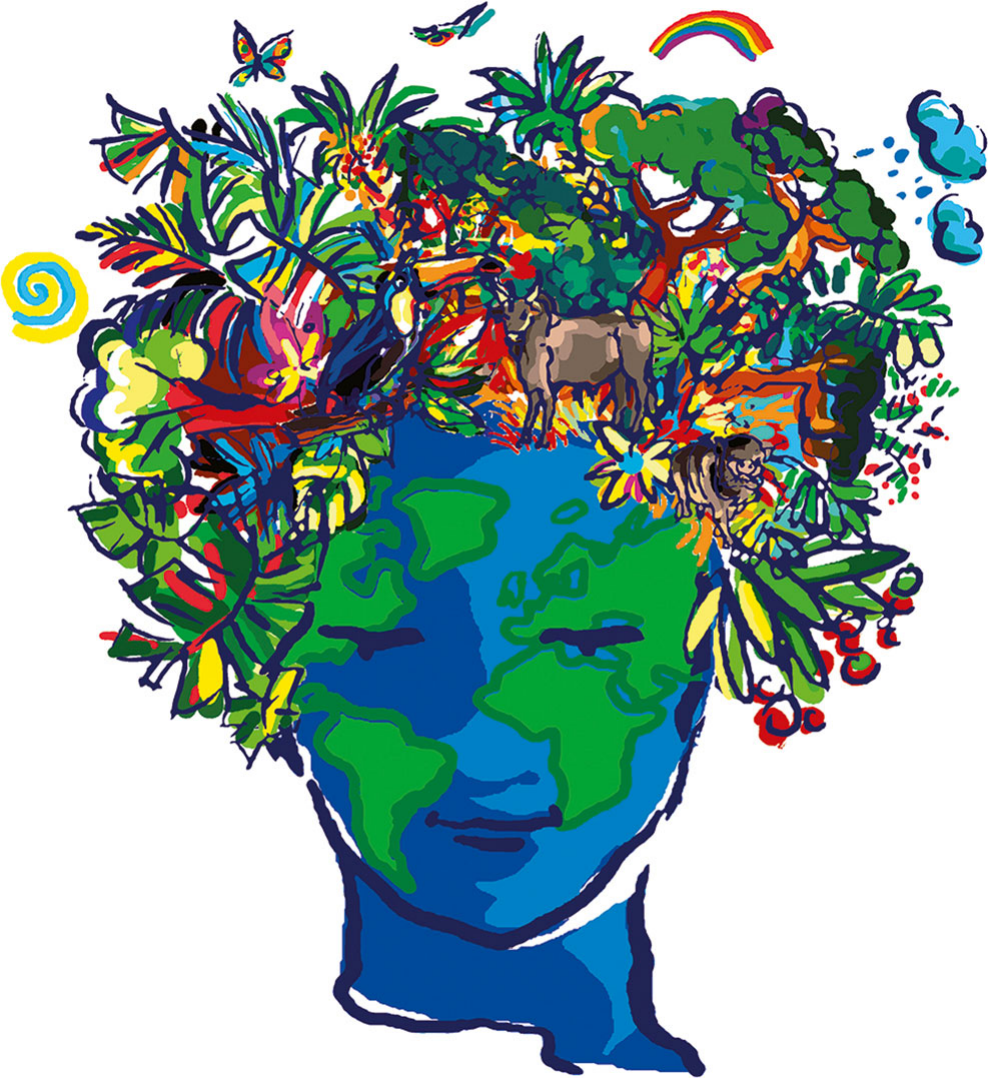
Vibrant and innovative local-specific human-driven systems as engine for a profound food system transformation (source: N. Le Gall/Cirad—Année international des Forêts 2011). Illustrates the profound food system transformation that is required to achieve the 2030 Agenda on Sustainable Development and the Paris Agreement on Climate and that is made of four parts (nutritious and healthy food, sustainable agricultural production and food value chains, mitigation of climate change and resilience, renaissance of rural territories). Such a transformation relies on the capacity to design and implement local specific innovation based initiatives to address local and national expectations through diverse adapted pathways. It also depends on the capacity to stimulate such initiatives and to orchestrate such a transformation at the global level to ensure orientation and consistency among scales

In a world increasingly focused on sustainable futures for people and planet, there is growing recognition of the important role played by agriculture. Following the food price spikes in 2008, there was increased awareness of the multiple inter-relationships between agriculture and key social, environmental, and economic issues. The sector is less and less considered as a problem, and more and more is seen as a solution (Brussaard et al. 2010; Lipper et al. 2014). The 2030 Agenda for Sustainable Development offers new momentum for work on the potential contributions of agriculture to public goods. It can be viewed as a powerful lever for the achievement of the overall 2030 Agenda. For this to happen, there has to be a transformation of food systems as a whole and not only from a sectorial point of view throughout the world: this has four interdependent parts that must be initiated and managed. This applies despite the diversity of local contexts as illustrated by Fig. 1, the pathways being followed, and the solutions that are developed. It means taking account of trade, climate change, global health, ecosystems, migration, actions of corporations, and of global social movements: all these elements justify the use of a global framework.

### Healthy and sustainable food consumption patterns

The first part of the transformation relates to food consumption patterns. This challenge is amplified by the unprecedented questions raised by the supply of an increasing urban population. What must be produced in the future, both in terms of volume and quality and the social, health, and environmental footprints of production modes, will mainly depend on what is consumed, wasted, thrown away, or recycled. Unhealthy diet is now recognized as a universal problem and the number one risk factor driving the world’s disease burden (Forouzanfar et al. 2015). Malnutrition irreversibly prevents hundreds of millions of people from reaching their full potential capabilities of living a healthy and productive life and is broadly recognized as a social injustice. Food consumption is an important area of innovation and public policy due to its links with production, value chains, the environment, nutrition, and health (Porter et al. 2014). Sustainable and nutrition-sensitive food consumption patterns should be supported through favorable food environments (HLPE 2017b). Dietary changes and reductions in food wastage are core elements of the SDG for sustainable consumption and production (goal 12) and, more broadly, of all SDGs.

### A new vision of sustainable agricultural production and food value chains

The second part of the transformation involves the promotion of inclusive, sustainable, and nutrition-sensitive agricultural production, processing, distribution, and marketing. It should consider the multiple functions of, and demands made on, agriculture and food. Sustainable agriculture can create decent jobs, support inclusive growth, improve livelihoods, and adapt to climate change. It must be implemented in ways that are tailored to each context. FAO estimates suggest that the economic empowerment of rural women through an equal access to productive resources (reflected in SDG 1) could increase yields on their farms by 20–30%, lifting 100–150 million persons out of hunger (FAO 2011b).

None of these changes are attainable in the absence of healthy ecosystems and their associated services. The challenge is to increase agricultural production on existing agricultural lands in ways that ensure biodiversity, maintain the integrity of ecosystems, and sustain ecosystem services: it is one of the world’s core sustainability challenges. Patterns of agricultural production and the measures of agriculture’s performance and effects must be reconsidered in ways that take account of the multiple functions expected from agriculture, including adaptation to and mitigation of climate change, biodiversity management, the provision of ecosystem-based services, people’s incomes, and just societies.

Pioneer farmers are pursuing ecologically sound agricultural practices and are well able to contribute to this part of the transformation. Numerous technical advances have been developed and subjected to scientific analysis—including agroecology (Wezel et al. 2009; IPES-Food 2016) and organic agriculture (Halberg and Müller 2013). Agroecology—in its many incarnations—is now considered by many global leaders as an approach that can promote the transformation, as stated during a symposium organized by the FAO in April 2018.

One promising approach is to understand biological and ecological regulatory mechanisms and to amplify them to increase the efficiency of resource use in agricultural systems. This approach can help to design (and then assess) sustainable production systems: it involves the use of multiple criteria and many different measurement scales. It calls for the analysis and strengthening of systems for innovation: this is a challenge for scientists to better consider local knowledge and expertise (Hainzelin 2013; Caron et al. 2014; Tittonnell 2014). There is growing evidence regarding the impact of such approaches on increasing incomes and improving food and nutrition security (HLPE 2016, *op. cit*.), on improving the resilience of small-scale farms, and on reducing greenhouse gas emissions (FAO 2013, *op. cit*.). As this is an emerging area for scientific analysis, there are controversies about the potential impact of different production models and the best pathways to pursue in different settings. This leads to the co-existence of different—sometimes conflicting—narratives (Petersen and Snapp 2015). There is therefore a need for stimulating innovation that is adapted to each situation, which addresses barriers and obstacles and that generates impact at scale.

### Contributing to mitigate climate change

A concerted response to the challenge of climate change is at the heart of the 2015 Paris Agreement and is central to the 2030 Agenda for Sustainable Development. It is the third part of the sustainable transformation of food systems. The starting point is the contribution of agriculture and land use changes to GHG emissions and the limited capacity of existing agricultural and food systems’ practices to lower their climate footprint. Simply put, if climate action is to be effective, there must be profound changes to agricultural systems (Lipper et al. 2014, *op. cit*.).

Climate-compatible and sustainable agriculture, in particular Climate-Smart Agriculture (FAO 2013), identifies the synergies that can result from agricultural practices that mitigate emissions of greenhouse gases (and reduce emissions through carbon capture), strengthen the social-ecological resilience of agricultural landscapes and rural communities in the face of unpredictable weather patterns, and contribute to improvement in rural livelihoods through sustainable improvements in productivity. Thus, although farming and land use changes are important drivers of climate change, they can also contribute to reduced emissions (Lipper et al. 2014, *op. cit.*). Provided that opportunistic behavior, in particular green-washing, is avoided, transformed agriculture and food systems can be important levers for effective climate action.

### A renaissance of rural territories

The fourth part of sustainable food system transformation reflects the extraordinary potential for territory-based institutions to stimulate people’s well-being through providing a range of social, economic and environmental functions and services that are essential to the whole of society (OECD/FAO/UNCDF 2016). Effective action at territorial level contributes to the food and nutrition security of rural and urban populations, to steady and shared economic growth, to decent jobs for young people, and to reducing root causes of frustration and conflict, which can lead to unrest, violence, and forced migration (Mercandalli and Losch 2017). In practice, this requires the establishment of trusted means to encourage—among others—greater equality of opportunity including gender equity, the sustainable management of natural resources, resilience in the face of climate change, as well as access to clean air, to water and sanitation, to renewable energy sources including wind and solar radiation, and to telecommunications.

At the heart of vibrant territories are strong political institutions and a wealth of social capital—at the local as well as at national and regional levels. The institutions need to be strong enough to support food systems transformation (Rigg 2006). This requires people within territories being empowered to develop their visions for sustainable development and then to implement meaningful activities. This is particularly important in rural areas, since, after decades of public disinterest, rural areas and their inhabitants are at high risk of being left behind. When this happens, it has a negative impact on all dimensions of sustainable development. The alternative is a rural *renaissance*, in which the relationships between rural and urban populations are recrafted within a renewed rural–urban social contract. It is central to the achievement of the SDGs, and the alternative—a process of urbanization that is built on the deprivation of rural areas—is widely seen to be unsustainable and likely to drive the migration of people from rural areas to towns and cities (HLPE 2017a, *op. cit*.).

## The new food systems transformation

These four parts together make up the food systems transformation that is required if the SDGs are to be achieved. The use of the term “transformation” is deliberate as incremental change will not be enough. The breadth and depth of the transformation required suggest that it must be supported by people who are committed to radical, collective and long-term change. We do not refer to it as a revolution, since it must happen as a well-conceived and carefully planned process that engages all stakeholders. Considerable intellectual and material investment is required to make it happen. The investment should result in exploration of a broad range of options and should be explored as a basis for developing novel strategies and practices (Godfray et al. 2010, *op. cit*.). Barriers and obstacles that impede action must be identified and overcome. This includes power imbalances and conflicts of interest across food systems (HLPE 2017b), as well as the trade-offs needed to align local systems with global priorities for sustainability. Managing the trade-offs calls for enlightened governance and political arbitration. The investment includes an exceptional national and inter-national mobilization of people with the capability to do this work and to establish means to build inclusive, sustainable, and safe agriculture, food, and rural systems. The people who lead it must be able to embrace the four components of food systems transformation and to create optimal conditions for their implementation. The transformation will not occur spontaneously: it must be planned, designed, implemented, and monitored by those who will be locally involved in implementation working within agreed parameters for sustainable development at national and global levels.

The first stage of implementing the transformation depends on the existence of agreed orientations that are shared among the actors. Context-adapted goals need to exist at all levels, from local to global, that are fully in line with the SDGs. There will be tensions between different interests and handling them calls for political management of trade-offs between stakeholders and among processes of arbitration. A consistent framework is needed to promote the transformation while addressing the complex and interrelated challenges, particularly the synergies and trade-offs between what is expected at local, national and global level.

The second stage of implementing the transformation requires the involvement of scientific groups and political actors through enabling them to access new knowledge to learn new processes and to implement them intensively (Caron et al. 2014, *op. cit*.). In recent decades, agricultural innovation has tended to promote homogeneity and uniformity: the transformation recognizes the virtues of diversity and context-adapted solutions. “Context-adapted” and “place-based” solutions should be favored over “one size fits all” prescriptions (IAASTD 2009)—even if the latter maintain the illusion to be easily taken to scale.

The third stage of implementing the transformation relies on shifts in the governance of food systems so that they prioritize human development and people’s food and nutrition security, the stewardship of renewable resources, long-term ecosystem health, as well as equitable growth, trade, and consumption (Lambek et al. 2014). This requires the design of new policy frameworks at national—and global—levels. Such shifts can only take place if they are supported by all stakeholders—including businesses. While it is the role of Government to establish policy and define standards, the governance of food systems has to be both multi-scalar and multi-stakeholder, and this adds to complexity (Lang et al. 2009). It is important that the interests of the many people who are poor, vulnerable, and at risk of being left behind are prioritized.

Finally, the fourth stage of implementing the transformation relies on new ways of thinking, planning, and managing policies and programs for production, consumption, innovation, and rural development. Linear thinking and logic models that seek to prescribe results need to evolve into approaches that embrace complexity, focus on socio-political processes and transitions, take account of the multiple relationships between stakeholders, and consistently commit to the empowerment of all peoples in ways that enable them to realize their human rights (Ferrero and Zepeda 2014).

The four stages of implementing the transformation remind us that success results from multiple actions along a range of pathways. The transformation cannot be advanced through one universally applicable technical model: pathways must be context-specific, multi-dimensional, and integrated. Advancing the transformation requires the design and implementation of new and differentiated actions at local level, responding to expectations of different stakeholders, reflecting national policy, and—at the same time—seeking to impact at scale, so contributing to achievement of the SDGs. The affirmation of this plurality, like the uncertainties regarding the paths to follow in each place, reflects the pre-eminence given to local knowledge and innovations, including from farmers themselves, as well as the local application of scientific expertise. It will result in the revaluing of different forms of knowledge.

There are three prerequisites for successful implementation of the four-part transformation, which needs immediate attention. First, metrics that aid planning, implementation, and monitoring must be designed and tested. Second, links between local and global action must be organized to enable coherent changes on a significant scale. Third, territorial approaches must be used to incentivize actors so that they adopt new practices.

### Assessing contributions of food systems to the SDGs

To appreciate the contribution of food systems to the SDGs, we need (a) to be able to describe their characteristics with a common language and (b) to measure systems performance in relation to the SDGs. There is still much to be done on how to measure performance: this need is leading numerous authors to propose new methods and indices. The explosion of indices is unsurprising because of the wide range of issues involved. Many countries are already implementing multi-dimensional poverty measures (Alkire and Robles 2016). The International Food Policy Research Institute (IFPRI) has proposed a Food Security Index (http://ghi.ifpri.org/) to serve as a dashboard. More recently, FAO has developed the Food Insecurity Experience Scale: this has been adopted in the SDG indicator framework (FAO 2016c).

The articulation of the 17 SDGs requires us to completely modify how performance is conceived and measured. The UN Statistical Commission has developed an indicator framework for the SDGs that is expected to be further refined in the coming years to include some of these complex variables (UN 2015).

It will be critical for the food system transformation that the SDG indicators integrate the core variables that define the overall transformation and its four constituent parts. This calls for further work to ensure that frameworks and indicators can fully describe the nexus of food and nutrition security, environmental health, climate and social justice, as well as the impacts of food systems on the nexus. The frameworks and indicators must be applicable at local levels and—at the same time—contribute to analysis at global level. The metrics that derive from these frameworks should be publicly available so that those who make investments can assess the extent to which they succeed in transforming food systems and to which they will contribute to change at scale.

**Fig. 2:**
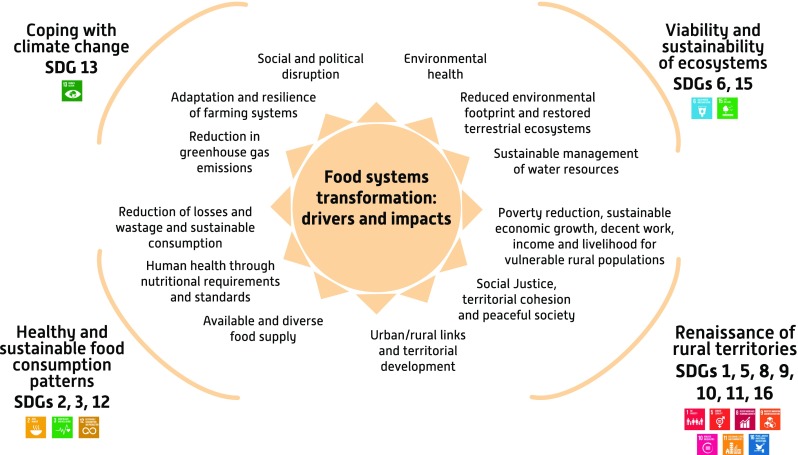
Assessing the food systems transformation capacity to address the Agenda 2030 through the agriculture–food and nutrition security–environment health–climate–social justice nexus. Suggests a general framework for food systems transformation by highlighting the four parts, each of which can be characterized with specific variables. These can be used to design relevant indicators for assessing the impact of system transformation

We propose a framework that has two overarching characteristics. First, it takes interactions between food and nutrition security, environmental health, climate, and social justice into account. Second, it focuses on ways in which the nexus is influenced by changes in food systems. We believe that the framework can help with identifying potential indicators and developing them. The combination of framework and indicators should encourage the production of evidence that can support policy decisions and action in different contexts. The framework is described in Fig. 2.

### Achieving impact at scale through local-level action

The combination of research-based innovation and traditional knowledge yields multiple options for transforming food systems at the local level. For example, many rural communities permanently adapt agricultural practices so that their livelihoods can become more resilient in the face of climate change. Local-level change contributes to the overall transformation of food systems. Ideally, this local knowledge and experience should be made more widely available so as to examine the extent to which they can be applied more widely across nations and regions. Yet, most of these local experiences are not directly reproducible in different agricultural–sociological–economic systems and this limits the extent to which they can be taken to scale. This suggests that the transformation of food systems cannot rely exclusively on universal approaches or the scaling-up of local specific solutions.

The food system in each locally setting is characterized by context-specific environmental, cultural, and agronomic features. There may also be locally specific patterns of financial investment and of trade. The local system is influenced by its interactions with large-scale processes, such as specific consumer demand, via the retail sector. The local system functions in ways that reflect the interests of the agriculture and food sector: these are determined by power relations and social structures and are affected by long-standing agreements, trade-offs, and conflicts. Because of the heterogeneity of local food systems, and the ways in which they are shaped by the contexts within which they operate, it is necessary to establish context-specific and localized pathways for transformation. In order to design and implement such pathways, it is the responsibility from political bodies and associated institutions to establish legitimate relevant objectives, assessment metrics, and indicators for food system transformation.

The large-scale impact of local food systems changes does not just result from the summation of local-level initiatives and processes: it depends on the simultaneous application of interventions at different levels. To assess the contribution of local changes to global impact, two other types of metrics will be needed.

The first is to quantify interaction between the different levels of intervention that contribute to system transformation (Gunderson and Holling 2002). This is needed because successful transformation depends on the successful integration of local and regional policies and initiatives. This includes the resolution of tension and sometimes conflicts between internationally agreed goals and local realities, interests, agreements, and habits. This involves effective governance and political arbitration in the event of trade-offs.

The second is to quantify the degree to which frameworks are being applied to support (a) policy coherence between local and national levels and (b) the management of trade-offs and compromises required to make coherence happen. One issue that has been highlighted is the intersection between local agriculture and food systems with the globalized market. This is critical—and quite controversial—given the concentration of food corporations, the homogenization of markets, and the expansion of international trade in food associated with the multiplication of international trade agreements. “Many economists argue that the environmental and social concerns associated with freer trade are best addressed with domestic policies that do not distort trade… Others encourage markets but support market interventions …, while the food sovereignty movement argues that local markets are the priority” (HLPE 2017a, *op. cit*.). The multi-stakeholder United Nations Committee on World Food Security should be sufficiently empowered by its members to provide global-level political governance for food systems transformation.

### Managing the intersection of global and local priorities through territorial approaches

A territory is much more than an administrative area. It is a bounded space that has stood the test of time, is owned by a social group that identifies with it, and which accepts specific forms of governance and control (Caron et al. 2017). A territory offers its inhabitants a form of social regulatory capacity that has been established at the interface between collective action and public administration: that latter represents an increasing commitment to sustainability. In practice, territories may be defined in different ways: a municipality or a coalition of municipalities, a traditional area for indigenous people, habitations around a watershed, a value-chain corridor, a production basin, and so on. Its people have a similar vision of their destiny and common concerns about the threats they face. They support the rule-based administration of public affairs and favor strong and well-directed collective action: this can buffer the impact of market and state failures on the territory and its people (Ostrom 1990). It usually involves a link between collective action and public administration, with efforts to resolve contradictions between them.

When food systems are transformed in ways that encourage resilience in the face of adverse weather, they bring multiple benefits both to people and to landscapes (Scherr et al. 2012). Such changes are dependent on the effective organizations of individual territories, on constructive relationships between rural and urban areas, among territories, with national authorities and with international institutions. Implementing a territorial approach to food systems (Benoît et al. 2006) involves working in five dimensions: (i) establishing an operational definition for what is meant by a territory; (ii) creating functioning institutions and governance platforms through building social capital and empowering local stakeholders; (iii) encouraging improvements in production through better rural infrastructure, links to markets, climate-compatible agriculture, and stimuli for non-farm economy; (iv) providing support for poorer people including safety-nets, conditional cash transfer programs, and other forms of social protection; as well as (v) implementing of territorial development as a national strategy through rural development policies and financing instruments.

## Conclusion

Inclusive and sustainable food systems are necessary not only for achieving SDG 2 but also as a contribution to the whole of the 2030 Agenda for Sustainable Development. Sustainable food systems may contribute to four outcomes: (i) enabling all people to eat nutritious and healthy diets, (ii) regenerating ecosystems, (iii) mitigating climate change, and (iv) encouraging social justice through focusing on the resilience and well-being of poorer rural communities. There are economic and political interests which will influence the realization of these outcomes: transformation efforts will be contested and need strong political support, including from within urban areas, if they are to succeed.

Vibrant rural territories, within which people produce food, deliver essential services, and contribute to the whole of the society, are indispensable. The SDGs will simply not be achieved without rural prosperity. The interdependence of rural and urban areas should be recognized and form the basis of a new rural–urban social contract. This will be the basis of society remunerating rural dwellers and their territories both for the functions they perform and for the public goods they provide to societies, the planet, and economies. To this end, it is important that relevant metrics are used to illustrate the benefits of sustainable, inclusive, and resilient food systems.

Although the pathways for most food systems changes are designed at local and national levels, the universal implementation of this four-part transformation should be pursued in global forums and advocated within global governance processes. The four parts should be mainstreamed in existing institutions, agreements, and conventions, in particular the UNFCCC. The newly established Koronivia joint work for agriculture might provide a relevant space within the process where analyses, metrics, knowledge platforms and learning could be encouraged. The four part transformation should feature strongly within national policies, societal norms, integrated management of territories and systems for public accountability. All actors, whatever their modes of production and consumption, should be encouraged to engage. There are no universal technical “fixes” for such a food systems transformation: the approach must always be adapted to the specificities of different locations. This means encouraging analyses, metrics, knowledge platforms, and learning that are locally relevant in ways that include all stakeholders.

There is always more to be learnt about the links between agriculture, climate, food and nutrition security, ecosystem regeneration, and social justice, given the constant evolution of humanity and the planet. Science is invited to help understand the links and the ways in which change is taking place over time and to enable decision makers to anticipate and appreciate what was not known before. Implementing the food systems transition will be knowledge intensive. But knowledge generation has a cost; it calls for well-directed investment in research that does not only deliver technology but also helps with understanding of dynamics, transitions, and interfaces. The research should help to decode each nexus, use metrics, quantify progress, and dissect out the basis of any disagreement. It should contribute to explore possible futures through foresight analysis, the identification of critical and emerging issues and to the formulation of policies.

The food systems transformation depends on enlightened policies, well-adapted processes, local to global integration, and value systems based on justice and human rights principles for arbitrating trade-offs. All concerned will need to think in interconnected ways that link systems, use novel data sets, and aid decision-making. These are substantial demands, but unless such changes are made, the transformation will not succeed. The experience of the Milano Group demonstrates how regular interactions among diverse leaders from different stakeholders—decision makers, implementers, scientists, farmers, civil society organizations, businesses, and consumers—can develop new narratives and result in collective action for transformation.

The process could be accelerated through multi-stakeholder coalitions to encourage greater alignment among actors in the framework of the UN system action as in the Committee on World Food Security, through encouraging science-based systems changes, curated Food Systems Dialogues, and high-level advocacy such as a global panel for Food Systems Transformation. This could follow the example of the Global Commission on the Economy and Climate led by former President of Mexico Felipe Calderón. Such a concerted effort for food systems transformation is key to the implementation of the Paris Agreement on Climate and the 2030 Agenda. It will also be an opportunity for the diverse actors that share a common vision to explore the links between evidence and policy and between local and global processes.
